# Long-Term Outcomes and Risk of Pancreatic Cancer in Intraductal Papillary Mucinous Neoplasms

**DOI:** 10.1001/jamanetworkopen.2023.37799

**Published:** 2023-10-17

**Authors:** Jaime de la Fuente, Arjun Chatterjee, Jacob Lui, Avinash K. Nehra, Matthew G. Bell, Ryan J. Lennon, Blake A. Kassmeyer, Rondell P. Graham, Hiroki Nagayama, Phillip J. Schulte, Karen A. Doering, Adriana M. Delgado, Santhi Swaroop Vege, Suresh T. Chari, Naoki Takahashi, Shounak Majumder

**Affiliations:** 1 Division of Gastroenterology and Hepatology, Mayo Clinic, Rochester, Minnesota; 2 Department of Internal Medicine, Cleveland Clinic, Cleveland, Ohio; 3 Department of Internal Medicine, Columbia University Irving Medical Center and the Vagelos College of Physicians and Surgeons, New York, New York; 4 Department of Radiology, Mayo Clinic, Rochester, Minnesota; 5 Department of Internal Medicine, Mayo Clinic, Rochester, Minnesota; 6 Department of Quantitative Health Sciences, Mayo Clinic, Rochester, Minnesota; 7 Department of Laboratory Medicine and Pathology, Mayo Clinic, Rochester, Minnesota; 8 Department of Gastroenterology and Hepatology, University of Texas MD Anderson, Houston

## Abstract

**Importance:**

Intraductal papillary mucinous neoplasms (IPMNs) are pancreatic cysts that can give rise to pancreatic cancer (PC). Limited population data exist on their prevalence, natural history, or risk of malignant transformation (IPMN-PC).

**Objective:**

To fill knowledge gaps in epidemiology of IPMNs and associated PC risk by estimating population prevalence of IPMNs, associated PC risk, and proportion of IPMN-PC.

**Design, Setting, and Participants:**

**:** This retrospective cohort study was conducted in Olmsted County, Minnesota. Using the Rochester Epidemiology Project (REP), patients aged 50 years and older with abdominal computed tomography (CT) scans between 2000 and 2015 were randomly selected (CT cohort). All patients from the REP with PC between 2000 and 2019 were also selected (PC cohort). Data were analyzed from November 2021 through August 2023.

**Main outcomes and Measures:**

CIs for PC incidence estimates were calculated using exact methods with the Poisson distribution. Cox models were used to estimate age, sex, and stage–adjusted hazard ratios for time-to-event end points.

**Results:**

The CT cohort included 2114 patients (1140 females [53.9%]; mean [SD] age, 68.6 [12.1] years). IPMNs were identified in 231 patients (10.9%; 95% CI, 9.7%-12.3%), most of which were branch duct (210 branch-duct [90.9%], 16 main-duct [6.9%], and 5 mixed [2.2%] IPMNs). There were 5 Fukuoka high-risk (F-HR) IPMNs (2.2%), 39 worrisome (F-W) IPMNs (16.9%), and 187 negative (F-N) IPMNs (81.0%). After a median (IQR) follow-up of 12.0 (8.1-15.3) years, 4 patients developed PC (2 patients in F-HR and 2 patients in F-N groups). The PC incidence rate per 100 person years for F-HR IPMNs was 34.06 incidents (95% CI, 4.12-123.02 incidents) and not significantly different for patients with F-N IPMNs compared with patients without IPMNs (0.16 patients; 95% CI, 0.02-0.57 patients vs 0.11 patients; 95% CI, 0.06-0.17 patients; *P* = .62). The PC cohort included 320 patients (155 females [48.4%]; mean [SD] age, 72.0 [12.3] years), and 9.8% (95% CI, 7.0%-13.7%) had IPMN-PC. Compared with 284 patients with non-IPMN PC, 31 patients with IPMN-PC were older (mean [SD] age, 76.9 [9.2] vs 71.3 [12.5] years; *P* = .02) and more likely to undergo surgical resection (14 patients [45.2%] vs 60 patients [21.1%]; *P* = .003) and more-frequently had nonmetastatic PC at diagnosis (20 patients [64.5%] vs 130 patients [46.8%]; *P* = .047). Patients with IPMN-PC had better survival (adjusted hazard ratio, 0.62; 95% CI, 0.40-0.94; *P* = .03) than patients with non-IPMN PC.

**Conclusions and Relevance:**

In this study, CTs identified IPMNs in approximately 10% of patients aged 50 years or older. PC risk in patients with F-N IPMNs was low and not different compared with patients without IPMNs; approximately 10% of patients with PC had IPMN-PC, and they had better survival compared with patients with non-IPMN PC.

## Key Points

**Question:**

What is the estimated population prevalence of intraductal papillary mucinous neoplasms (IPMNs), associated pancreatic cancer (PC) risk, and proportion of malignant transformation of IPMNs to PC?

**Findings:**

In this cohort study of 2114 patients aged 50 years or older, the prevalence of IPMNs on contrast-enhanced computed tomography imaging was 10.9%, more than 80% of which were branch-duct IPMNs with no worrisome or high-risk features, and the risk of PC and overall survival in patients with IPMNs without worrisome or high-risk features was similar to that of patients without IPMNs. Approximately 10% of PCs arose from IPMNs, and patients with these cancers had better outcomes.

**Meaning:**

This study found that PC risk was low in patients with Fukuoka-negative IPMNs and similar to that of patients without IPMNs and that while IPMN-associated PC accounted for a small proportion of all PC cases, these patients had better survival.

## Teaser

This cohort study investigates the prevalence of intraductal papillary mucinous neoplasms, their association with pancreatic cancer risk, and proportion with malignant transformation.

## Introduction

Incidental detection of pancreatic cystic lesions (PCLs) has increased in recent years primarily due to improved resolution and widespread use of cross-sectional imaging.^1,2^ Intraductal papillary mucinous neoplasms (IPMNs) are the most frequently identified PCLs.^3,4,5^ Current international consensus guidelines^4^ for management of IPMNs recommend image-based surveillance with the aim to detect clinical and imaging features of advanced neoplasia (high-grade dysplasia or pancreatic cancer). Although a minority of IPMNs warrant surgical resection, our existing knowledge about the prevalence, natural history, and risk of malignancy in IPMNs is largely derived from surgical series^6,7^ or from patients treated at tertiary centers,^8,9,10^ populations which are not representative of the population burden of the disease.^2,10,11^ There is a critical need to define the prevalence and natural history of IPMNs in a population-based cohort to inform surveillance guidelines.

A 2019 systematic review and meta-analysis^12^ estimated the pooled population prevalence of PCLs at approximately 8%. The reported prevalence varies across studies depending on the imaging modality used, geographical location, and age of the cohort studied, and most studies include all PCLs not specifically IPMNs. Furthermore, to our knowledge, there are no population-based estimates of the burden of pancreatic cancer (PC) in individuals with IPMNs or the proportion of PCs that develop from or adjacent to an IPMN. This study uses a population-based cohort to assess the prevalence and associated long-term outcomes and PC risk in IPMN. In addition, we investigated the proportion of PCs that arose from IPMNs (IPMN-PCs) and compared their outcomes with those of non-IPMN PCs.

## Methods

This cohort study was approved by the Mayo Clinic Foundation Institutional Review Board and Olmsted Medical Center Institutional Review Board (IRB). The IRB approved a waiver of the requirement to obtain informed consent in accordance with 45 CFR §46.116 as justified by the investigator and a waiver of Health Insurance Portability and Accountability Act of 1996 (HIPAA) authorization in accordance with applicable HIPAA regulations. The study is reported following the Strengthening the Reporting of Observational Studies in Epidemiology (STROBE) reporting guideline.

The Rochester Epidemiology Project (REP) is a medical records linkage system that provides longitudinal, population-based medical data for residents of Olmsted County, Minnesota. The REP database captures more than 95% of the medical care delivered to county residents across all community health care institutions and has served as a valuable resource facilitating population-based research in a variety of diseases.^13,14^ From the REP database, we created 2 population cohorts to address our aims: the computed tomography (CT) cohort to estimate the population prevalence and natural history of IPMNs and the PC cohort to investigate the proportion of PCs that arose from malignant transformation of IPMNs and compare clinical outcomes with those of non-IPMN PCs.

### CT Cohort

Using the REP database and procedure codes for abdominal imaging, we identified all unique Olmsted County residents between January 1, 2000, and December 31, 2015, aged 50 years or older who underwent a contrast-enhanced abdomen CT scan (eTable 1 in Supplement 1). We divided the 16-year study into 4-year periods and used a computer-generated, stratified random sampling of a total 2500 unique patients: 625 patients from each 4-year period. See exclusion criteria in eAppendix 1 in Supplement 1.

Clinical and demographic data were entered in the study database (eAppendix 2 in Supplement 1). Race was identified from the electronic health record by study team members (A.C. and J.L.). Race options in the databases were Black, Asian, White, other, and unknown. Information regarding race was collected given that this was a population study and the demographics of the population studied may not be generalizable to other populations. CT images were manually reviewed (J.D.L.F. and A.N) for each study individual. If new pancreatic findings or discrepancies from what was described in the original CT scan report were identified, images were reviewed by a second reviewer (H.N. or N.T.) to ensure accuracy. If a PCL was present, it was categorized as an IPMN (branch duct, mixed, or main duct) based on our a priori definitions (eAppendix 3 in Supplement 1).^9,15^ PCLs that met IPMN criteria were further classified as Fukuoka high-risk (F-HR), worrisome (F-W), or negative (F-N) based on current international consensus guidelines.^4^ If patients with IPMNs had subsequent cross-sectional imaging of the abdomen (contrast CT or magnetic resonance imaging [MRI]) performed more than 6 months from the date of index imaging, the most recent cross-sectional imaging and clinical data were also recorded.

### PC Cohort

Using the REP database, we identified all patients aged 18 years or older with confirmed PC in Olmsted County between January 1, 2000, and December 31. 2019. These patients were initially identified using diagnostic codes (eTable 2 in Supplement 1). Then, each patient electronic medical record was manually reviewed to ensure accurate diagnosis and determine patients who had pathology-confirmed pancreatic ductal adenocarcinoma or cancer that was clinically treated as PC based on imaging and laboratory testing. Patients who were not Olmsted County residents for 1 year or more were excluded. For each unique patient, we collected relevant demographic, clinical, and staging^16^ data (eAppendix 4 in Supplement 1).

The cross-sectional imaging at the time of PC diagnosis was reviewed by the study radiologist (N.T.). Each patient was classified using established a priori imaging criteria as definitive IPMN-PC, probable IPMN-PC, possible IPMN-PC, or non-IPMN PC (definitions of classifications used are in eAppendix 5 in Supplement 1). For patients in the PC cohort, surgical pathology was reviewed by a gastrointestinal pathologist (R.P.G.) to confirm histology and determine if the PC arose from an IPMN.

### Statistical Analysis

All data for our study were stored and managed with a research electronic data capture tool.^17^ Continuous data are summarized as mean (SD) unless otherwise noted. Categorical data are presented as frequency (percentage). CIs for binomial proportions were calculated using the Agresti-Coull method. CIs for incidence estimates were calculated using exact methods with the Poisson distribution. We used the 2-sample *t* test and Pearson χ^2^ test to compare independent groups for continuous and discrete variables, respectively. The Stuart-Maxwell test for marginal homogeneity was used to test for differences in discrete variables between baseline and follow-up images. Standardized incidence ratios (SIRs) were calculated to estimate the difference in future risk of PC between patients with and without IPMN cysts in the CT cohort. SIR was calculated by computing incidence rates within age (per year)–, sex-, and IPMN-specific cross-tabulations, then using a generalized linear model (log link) with age (3–degrees of freedom spline), sex, age-sex interaction, and IPMN as explanatory variables, with the log of person-years as an offset. Kaplan-Meier curves for PC risk in the CT cohort were produced using age as the time scale to account for the different age distribution between patients with and without IPMNs. Curves were compared using the score test from a Cox model, with age as the time scale and sex as a stratification variable. Cox models were used to estimate age, sex, and stage–adjusted hazard ratios (HRs) for time-to-event end points (eg, mortality or pancreatic cancer). An adjusted survival curve for IPMN-PC was calculated using estimated survival from the Cox model applied to an IPMN-PC cohort with the covariate distribution of the non-IPMN PC group. All hypothesis tests were 2-tailed with a .05 significance level. Analyses were conducted using R statistical software version 4.1 or higher (R Project for Statistical Computing). Data were analyzed from November 2021 through August 2023.

## Results

### CT Cohort

#### Prevalence of IPMNs

**Figure 1.**
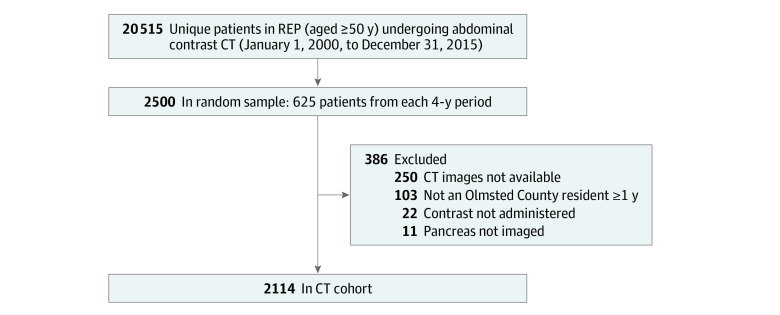
Study Flowchart of Computed Tomography (CT) Cohort REP indicates Rochester Epidemiology Project.

**Figure 2.**
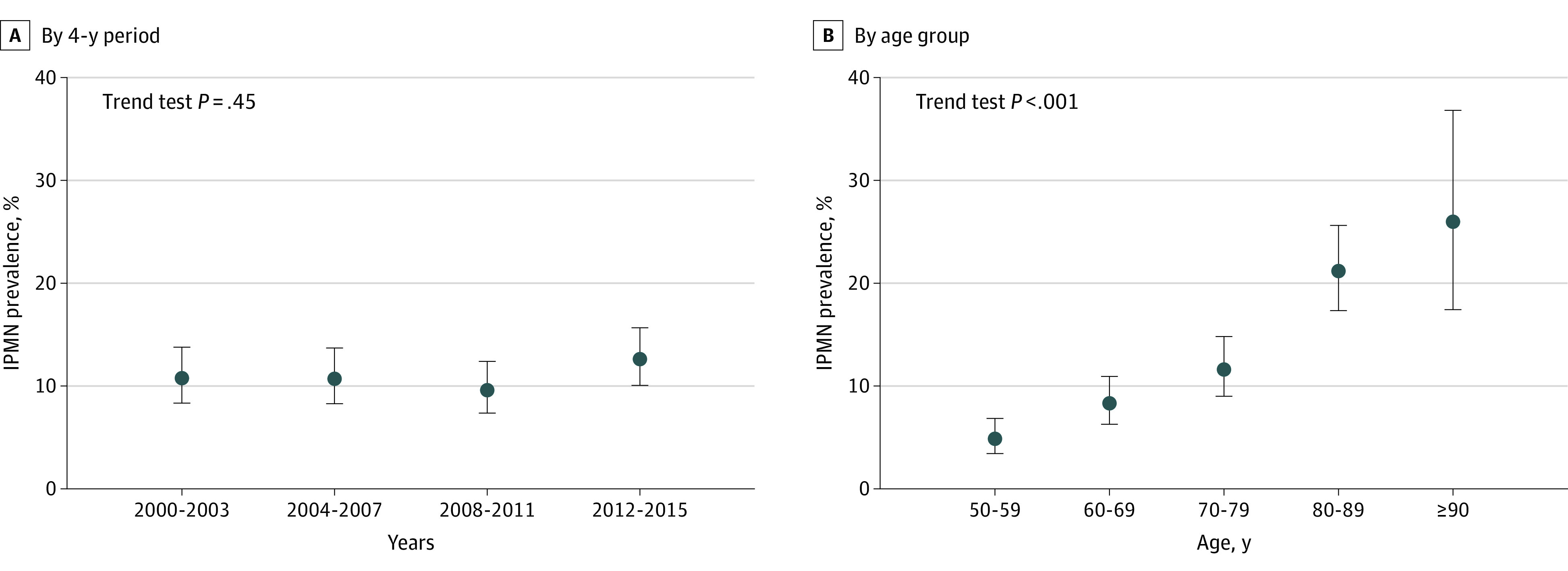
Prevalence of Intraductal Papillary Mucinous Neoplasms (IPMNs) The prevalence of IPMNs using computed tomography is presented A, in each 4-year period of the study and B, in age group by decade.

A total of 2114 patients in the CT cohort (55 Asian [2.6%], 42 Black [2.0%], 1943 White [91.9%], and 74 other race or race not available [3.5%]; 1140 females [53.9%] and 974 males [46.1%]; mean [SD] age, 68.6 [12.1] years) met study eligibility criteria (Figure 1). Of the CT cohort, 231 patients (10.9%; 95% CI, 9.7%-12.3%) were found to have an IPMN while 1883 patients (89.1%; 95% CI, 87.7%-90.3%) did not have an IPMN. Of 231 IPMNs, 80 IPMNs (34.6%) were described in the original radiology report while 151 IPMNs (65.4%) were found after independent review. This prevalence was consistent over the four 4-year periods (Figure 2A). The likelihood of discovering an IPMN increased with age, from 4.9% (95% CI, 3.4%-6.8%) in patients aged 50 to 59 years to 8.3% (95% CI, 6.3%-10.9%) in those aged 60 to 69 years, 11.6% (95% CI, 9.0%-14.8%) in those aged 70 to 79 years, 21.2% (95% CI, 17.3%-25.6%) in those aged 80 to 89 years, and 26.0% (95% CI, 17.4%-36.8%) in those aged 90 years or older (Figure 2B). Patients with an IPMN compared with patients without an IPMN were older (mean [SD] age, 75.4 [12.1] years vs 67.7 [11.8] years; *P* < .001) and more often female (147 females [63.6%] vs 993 females [52.7%]; *P* = .002) and had a lower median (IQR) body mass index (BMI; calculated as weight in kilograms divided by height in meters squared; 26.0 [23.1-29.3] vs 27.7 [24.2-32.1]; *P* = .002). Race, smoking history, jaundice, history of acute pancreatitis, family history of pancreatic cancer, diabetes history, serum cancer antigen 19-9 (CA 19-9) level, fasting blood glucose level, and hemoglobin A_1c_ level were similar in the 2 groups (eTable 3 in Supplement 1).

Most IPMNs (210 IPMNs [90.9%]) were branch duct, while 16 IPMNs (6.9%) were main duct and 5 IPMNs (2.2%) were mixed. By Fukuoka criteria,^4^ 187 IPMNs (81.0%) were F-N, 39 IPMNs (16.9%) were F-W, and 5 IPMNs (2.2%) were F-HR. Overall in the CT cohort, 5 patients (0.23%) had an F-HR IPMN. Of 187 F-N cysts, all were branch duct, whereas of 44 F-HR and F-W IPMNs, 16 cysts (36.4%) were main duct and 5 cysts (11.4%) were mixed. The most frequent high-risk and worrisome features detected were obstructive jaundice (4 patients) and a main pancreatic duct (MPD) diameter of 5 to 9 mm (22 patients), respectively. Most IPMNs were solitary (140 IPMNs [60.6%]), and of 217 IPMNs with location identified, they were more frequently located in the head (84 IPMNs [38.7%]) and tail (73 IPMNs [33.6%]) compared with the body (45 IPMNs [20.7%]) and neck (15 IPMNs [6.9%]) (*P* < .001 for equal proportions). The mean (SD) size of the largest cyst was 8.8 (5.5) mm. The population prevalence of a cyst greater than 3 cm in diameter was 3 patients (0.1%). Of 217 IPMNs with size measured, 148 cysts (68.2%) were less than 10 mm in diameter. Of 21 IPMNs with a dilated MPD, the mean (SD) MPD diameter was 6.2 (1.3) mm; the population prevalence of an MPD diameter greater than 9 mm was 4 patients (0.2%).

#### Natural History of IPMNs

Among patients with IPMNs, 107 individuals (46.3%) received follow-up with at least 1 abdominal cross-sectional imaging (CT or MRI) more than 6 months after the initial contrast CT scan, with a median (IQR) interval of 5.3 (2.2-6.2) years between index CT scan and latest abdominal imaging. A mean (SD) increase in maximal cyst diameter of 1.6 (4.7) mm was noted in 44 patients (41.1%), and 15 patients (14.0%) had additional new cysts identified on follow-up imaging. There were 6 patients (5.6%) who progressed within Fukuoka criteria. Of 86 patients who were F-N at baseline, 6 patients (6.9%) progressed to F-W (4 patients) or F-HR (2 patients). Patients who progressed from F-N to F-HR did so within 5 years of the index CT scan. No F-W IPMNs progressed to F-HR.

**Figure 3.**
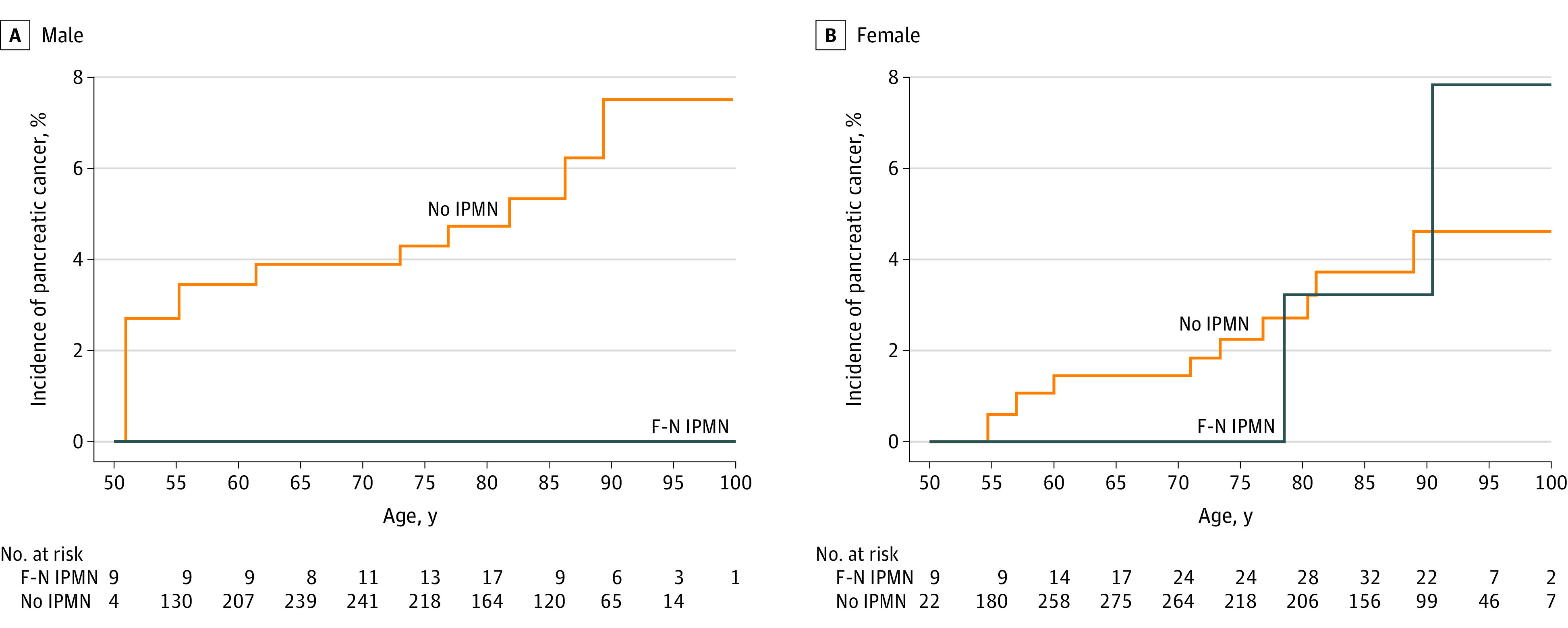
Incidence of Pancreatic Cancer Incidence curves for males and females of pancreatic cancer between the Fukuoka-negative (F-N) intraductal papillary mucinous neoplasm (IPMN) population and non-IPMN population. Patients entered the at-risk set according to their age at computed tomography.

In 231 patients with IPMNs, there were 4 PC diagnoses during a median (IQR) follow-up of 12.0 (8.1-15.3) years and 1507 total person-years (2 of 5 patients in F-HR and 2 of 187 patients in F-N). In 1883 patients without IPMNs, there were 17 PC diagnoses during a median (IQR) follow-up of 11.7 (8.4-16.4) years and 15 656 total person-years. Patients with F-N IMPNs who developed PC during surveillance progressed to F-HR, and they both developed PC within 5 years of the index CT scan. The PC incidence rates per 100 person-years for F-HR, F-W, F-N, and non-IPMN groups were 34.06 incidents (95% CI, 4.12-123.02 incidents), less than 0.01 incidents (95% CI, <0.00-1.56 incidents), 0.16 incidents (95% CI, 0.02-0.57 incidents), and 0.11 incidents (95% CI, 0.06-0.17 incidents), respectively (*P* < .001). Incidence rates per 100 person-years were not significantly different for patients with F-N IPMNs compared with patients without IPMNs (0.16 incidents; 95% CI, 0.02-0.57 incidents vs 0.11 incidents; 95% CI, 0.06-0.17 incidents; *P* = .62). Overall, the risk of developing PC during follow-up was not significantly different in patients with F-N IPMNs (SIR = 1.26; 95% CI, 0.28-5.56; *P* = .76) compared with individuals without IPMNs (Figure 3). The overall survival in these groups was similar (adjusted HR [aHR], 1.05; 95% CI, 0.86-1.28; *P* = .64) after adjusting for age and sex. Even comparing all patients with non–F-HR IPMNs with patients without IPMNs, the risk of PC (SIR = 1.03; 95% CI, 0.23-4.55; *P* = .97) and the overall survival (aHR, 1.06; 95% CI, 0.88-1.27; *P* = .57) were similar after adjusting for age and sex. This cohort of 212 patients with non–F-HR IPMNs consisted primarily of patients with IPMNs less than 10 mm in diameter (145 patients [68.4%]), and IPMNs between 10 and 20 mm (57 patients [26.9%]) and 20 mm or greater (10 patients [4.7%]) were in the minority.

### PC Cohort

#### Prevalence and Outcomes of IPMN-Associated PC

**Figure 4.**
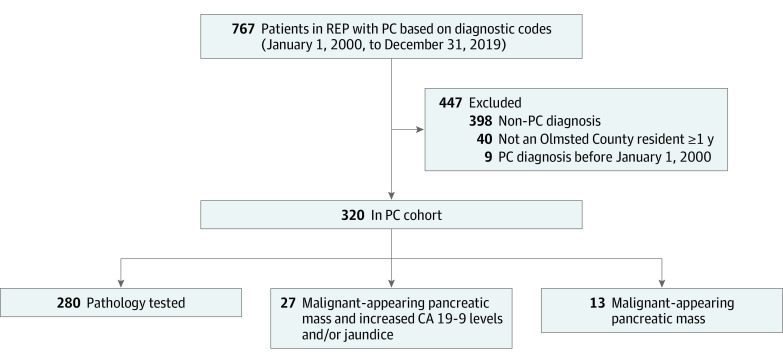
Study Flowchart of Pancreatic Cancer (PC) Cohort CA indicates cancer antigen; REP, Rochester Epidemiology Project.

The PC cohort included a total of 320 patients (6 Asian [1.9%], 4 Black [1.2%], 297 White [92.8%], and 13 other race or race not available [4.1%]; 155 females [48.4%] and 165 males [51.6%]; mean [SD] age at time of PC diagnosis, 72.0 [12.3] years) (Figure 4). At PC diagnosis, this population had a median (IQR) serum CA 19-9 level of 351 (44-2515) IU/L and mean (SD) BMI of 26.3 (5.8) and 18 patients (5.7%) had at least 1 first-degree relative with PC. At the time of diagnosis, clinical stage distribution was as follows: 71 patients with stage I (22.2%), 30 patients with stage II (9.4%), 49 patients with stage III (15.3%), 168 patients with stage IV (52.5%), and 2 patients with unknown stage (0.6%) cancer. The median overall survival was 7.0 months (95% CI, 5.5-8.0 months), with significantly longer survival among patients with earlier stage at diagnosis (stage I: 24.4 months; 95% CI, 20.1-40.9 months; stage II: 10.3 months; 95% CI, 7.5-41.2 months; stage III: 7.6 months; 95% CI, 5.6-10.1 months; stage IV: 3.3 months; 95% CI, 2.5-4.8 months; *P* < .001).

**Figure 5.**
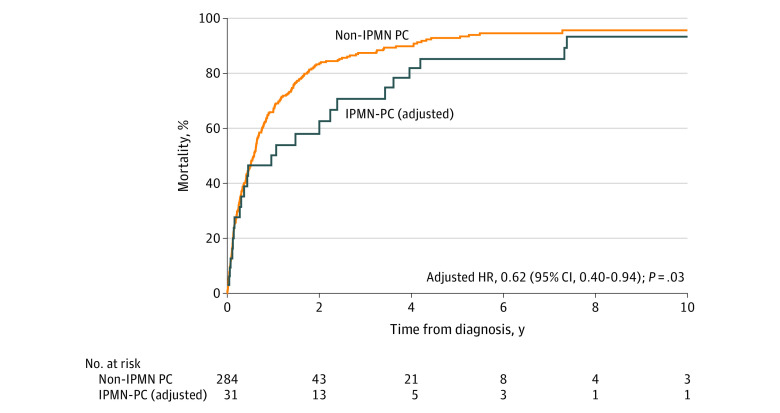
Adjusted Survival in Pancreatic Cancer (PC) With and Without Intraductal Papillary Mucinous Neoplasm (IPMN) Age, sex, and stage–adjusted survival in PC arising from IPMNs (IPMN-PC) and non-IPMN PC is presented. HR indicates hazard ratio.

Of 74 patients with PC who underwent surgical resection, 14 individuals (18.9%) were confirmed to have IPMN-PC after pathology expert review. Of patients with radiologic criteria for IPMN-PC, 11 individuals (3.5%) met criteria for definitive IPMN-PC, 15 individuals (4.8%) for probable IPMN-PC, and 21 individuals (6.7%) for possible IPMN-PC, while 266 individuals (85.0%) had non-IPMN PC. Combining patients with pathology-confirmed IPMN-PC and those without pathology confirmation but either definitive or probable IPMN-PC based on imaging, the prevalence of IPMN-PC was 31 patients (9.8%; 95% CI, 7.0%-13.7%). This prevalence of IPMN-PC decreased to 21 patients (6.7%; 95% CI, 4.4%-10.0%) if only definitive IPMN-PC was included from the imaging group. Including only individuals with cross-sectional imaging, surgical pathology, or both, 31 patients with IPMN-PC were older (mean [SD] age, 76.9 [9.2] vs 71.3 [12.5] years; *P* = .02), more likely to undergo surgical resection (14 patients [45.2%] vs 60 patients [21.1%]; *P* = .003), more-frequently had nonmetastatic cancer at diagnosis (20 patients [64.5%] vs 130 patients [45.8%]; *P* = .047), and had significantly improved age, sex, and stage–adjusted survival (aHR, 0.62; 95% CI, 0.40-0.94; *P* = .03) compared with 284 patients with non-IPMN PC (Figure 5). Demographic and clinical data for patients with IPMN-PC and non-IPMN PC are summarized in eTable 4 in Supplement 1.

## Discussion

This cohort study found that the estimated population prevalence of IPMNs on CT in individuals aged 50 years or older was 10.9% and this prevalence increased with age. Most of these were branch-duct IPMNs, and fewer than 20% of IPMNs had worrisome or high-risk features. During extended follow-up, development of IPMN-PC was infrequent and the PC risk in patients with IPMNs without worrisome or high-risk features was similar to that of individuals without IPMNs. We further identified that approximately 10% of PCs developed in the background of an IPMN. Compared with patients with non-IPMN PC, individuals with IPMN-PC were less likely to have metastatic disease at diagnosis and had improved overall survival.

This study focused on individuals aged 50 years or older given that PCLs are less likely to be incidentally detected in younger individuals and the risk of PC increases with age.^5,10,18,19,20^ We also specifically assessed IPMNs given that IPMNs are the most prevalent PCLs in clinical practice.^3,4,5^ We used contrast-enhanced CT scans given that this is the most widely used cross-sectional abdominal imaging modality in the US^1^ and is less impacted by selection bias compared with more pancreas-specific imaging modalities, such as MRI or endoscopic ultrasound. Previous studies^5,10,19,20^ using CT imaging reported a PCL prevalence of 1.2% to 5.4%. Our study identified a higher prevalence, likely associated with image review by experts and limiting of the study population to patients aged 50 years and older. Laffan et al,^10^ in a CT-based study in the US, described a PCL prevalence in individuals aged 50 to 89 years ranging from 1.5% from 8.7%, while our study found a prevalence of presumed or suspected IPMNs ranging from 4.9% to 21.2% for the same age group. This reported difference may be explained to a certain extent by our study selecting a random cohort from population-based data compared with the single tertiary referral institution in Laffan et al,^10^ which specifically excluded individuals with known pancreatic disease in an attempt reduce referral bias.

Multiple studies^8,9,15^ in tertiary centers have found that IPMNs were associated with increased PC risk and that risk was higher in the presence of F-W or F-HR features. Attempts to quantify the risk of malignant transformation of PCLs to PC using large administrative databases have found that overall risk was relatively low.^21,22^ Specifically, IPMN-PC was more frequently earlier stage at diagnosis and had overall improved survival compared with non-IPMN PC.^23,24^ Our population-based study’s findings agree with these prior observations. The improved prognosis of IPMN-PC has been attributed to an earlier stage of diagnosis and a greater proportion of nonmetastatic disease, as observed in our study. Moreover, the survival benefit in IPMN-PC has been primarily observed in node-negative disease, further highlighting outcomes associated with stage shift. Although several studies^8,9^ describe the prevalence of PC in patients with IPMN, the true prevalence in the general population has not been previously reported, to our knowledge. This study provides estimates of the prevalence of IPMN-PC in a population-based PC cohort and addresses a critical gap in our understanding of IPMN-PC epidemiology. Using our CT cohort, we also found that the risk of developing PC was relatively low. This is highlighted further in our study given that patients with F-N IPMNs did not have different rates of PC compared with patients without IPMNs. This finding highlights previous work published by our group finding that F-HR IPMNs were the primary contributors to the risk of developing PC while F-W and F-N IPMNs had a low 5-year PC risk.^15^ Although the risk of IPMN-PC is has been extensively described,^8,9^ our population-based study further demonstrates that most IPMNs did not progress in Fukuoka stage and did not transform into PC, a similar message expressed by the current American Gastroenterological Association pancreatic cyst guidelines, published in 2015,^25^ and studies published in 2022^26^ and 2016.^27^

### Limitations

We acknowledge several limitations in our study. The REP primarily represents a homogenous White population, and inferences from this data may not apply to a population that is more diverse. Most individuals in the CT cohort did not have pathology-confirmed IPMN. We used a priori imaging-based definitions to reduce risk of PCL misclassification.^9,15^ This is reflective of clinical management given that most IPMNs in clinical practice are presumed or suspected based on imaging and are not biopsy proven. The CT cohort was constructed randomly from a population-based sample of individuals who had undergone cross-sectional imaging. While this potentially introduced selection bias, it provided population-level data and improved on our current epidemiologic knowledge of IPMNs, which is primarily derived from hospital-based referral cohorts. While most CT scans in this population sample were performed for indications unrelated to the pancreas, we did not use indication for imaging as an inclusion or exclusion criterion; therefore, it is possible that some patients in this random sample underwent imaging for a known pancreatic cyst or suspected pancreas cancer. The alternative of an unbiased population-based imaging study to estimate incidental IPMN prevalence would be cost and effort prohibitive and expose study participants to the risk of contrast-enhanced imaging. Another possible limitation of our study was the use of CT instead of MRI. We selected CT imaging given that it is the most widely used imaging modality while recognizing that MRI is better at identifying small IPMNs. However, CT is generally able to detect IPMNs that are clinically relevant, and using MRI as the imaging modality could potentially limit the sample size. A similar population-based study using MRIs may be considered in the future and will be particularly relevant if assessing the incidence of IPMNs. A small subset of the IPMN population in this study underwent long-term imaging follow-up. While this limited the study sample size for assessing long-term outcomes associated with IPMNs, our study provides data on population-level outcomes in contrast to the more widely available referral center–based outcomes in this disease. The small number of PC events in the CT cohort resulted in wide CIs, and inferences based on asymptotic theory may be unreliable. Additionally, to detect IPMN-PCs, we combined imaging and pathologic criteria. Imaging criteria of IPMN-PCs have not been definitively established and may impact the accuracy of our prevalence estimates. However, these imaging criteria for IPMN-PCs were defined a priori in consultation with an expert pancreatic radiologist (N.T.) to avoid the inherent bias of limiting the study to surgically confirmed PC. To further address this limitation, we have included estimates in the subgroup of patients with surgically confirmed PCs.

## Conclusions

This cohort study provides population estimates exploring the association between IPMNs and PCs. In the general population, IPMNs on CT were present in approximately 1 of 10 patients aged 50 years or older, of which more than 80% were branch-duct IPMNs without high-risk or worrisome features. Among IPMNs that were F-N at baseline, fewer than 10% developed worrisome or high-risk features on follow-up. PC development in IPMN was a rare event overall. Moreover, in F-N IPMNs, the incidence of PC was not significantly different from that among patients without IPMNs, a key finding that if validated in a larger population could challenge the relevance of extended surveillance in this population of patients with F-N IPMNs who make up most of the IPMN population. We further found that malignant IPMNs comprised approximately 10% of PC diagnoses and that IPMN-PC was earlier stage at diagnosis and associated with improved survival regardless of stage at presentation compared with non-IPMN PC. Results of this study provide insights regarding IPMN prevalence and the associated PC risk and may inform future population surveillance strategies and guidelines for patients with IPMNs.

## Notes

Supplement 1. **eAppendix 1.** Computed Tomography Cohort Exclusion Criteria **eAppendix 2.** Clinical and Demographic Data of Computed Tomography Cohort **eAppendix 3.** Categories of Suspected and Presumed Intraductal Papillary Mucinous Neoplasm Based on A Priori Definitions **eAppendix 4.** Clinical and Demographic Data of Pancreatic Cancer Cohort **eAppendix 5.** Pancreatic Cancer Imaging Classification Using A Priori Definitions to Establish Malignant Transformation of Intraductal Papillary Mucinous Neoplasm **eTable 1.** Current Procedural Terminology Codes of Abdominal Computed Tomography (CT) Imaging Used to Develop CT Cohort **eTable 2.** Index Codes Used to Collect Pancreatic Cancer Cohort **eTable 3.** Demographics and Clinical Characteristics of Computed Tomography Cohort **eTable 4.** Demographics and Clinical Characteristics of Pancreatic Cancer Cohort

Supplement 2.
Data Sharing Statement
