# Supplementary material for: Long-Term Outcomes and Risk of Pancreatic Cancer in Intraductal Papillary Mucinous Neoplasms
Source: JAMA Netw Open. 2023 Oct 17;6(10):e2337799. doi: 10.1001/jamanetworkopen.2023.37799 (PMC10582793; doi:10.1001/jamanetworkopen.2023.37799)
Supplement: Supplement 1. — eAppendix 1. Computed Tomography Cohort Exclusion Criteria eAppendix 2. Clinical and Demographic Data of Computed Tomography Cohort eAppendix 3. Categories of Suspected and Presumed Intraductal Papillary Mucinous Neoplasm Based on A Priori Definitions eAppendix 4. Clinical and Demographic Data of Pancreatic Cancer Cohort eAppendix 5. Pancreatic Cancer Imaging Classification Using A Priori Definitions to Establish Malignant Transformation of Intraductal Papillary Mucinous Neoplasm eTable 1. Current Procedural Terminology Codes of Abdominal Computed Tomography (CT) Imaging Used to Develop CT Cohort eTable 2. Index Codes Used to Collect Pancreatic Cancer Cohort eTable 3. Demographics and Clinical Characteristics of Computed Tomography Cohort eTable 4. Demographics and Clinical Characteristics of Pancreatic Cancer Cohort [file jamanetwopen-e2337799-s001.pdf]

Supplemental Online Content

de la Fuente J, Chatterjee A, Lui J, et al. Long-term outcomes and risk of pancreatic cancer with intraductal papillary mucinous neoplasms. *JAMA Netw Open*. 2023;6(10):e2337799. doi:10.1001/jamanetworkopen.2023.37799

eAppendix 1. Computed Tomography Cohort Exclusion Criteria

eAppendix 2. Clinical and Demographic Data of Computed Tomography Cohort

eAppendix 3. Categories of Suspected and Presumed Intraductal Papillary Mucinous Neoplasm Based on A Priori Definitions

eAppendix 4. Clinical and Demographic Data of Pancreatic Cancer Cohort

eAppendix 5. Pancreatic Cancer Imaging Classification Using A Priori Definitions to Establish Malignant Transformation of Intraductal Papillary Mucinous Neoplasm

eTable 1. Current Procedural Terminology Codes of Abdominal Computed Tomography (CT) Imaging Used to Develop CT Cohort

eTable 2. Index Codes Used to Collect Pancreatic Cancer Cohort

eTable 3. Demographics and Clinical Characteristics of Computed Tomography Cohort

eTable 4. Demographics and Clinical Characteristics of Pancreatic Cancer Cohort

This supplemental material has been provided by the authors to give readers additional information about their work.

© 2023 de la Fuente J et al. *JAMA Network Open.*

eAppendix 1. Computed tomography cohort exclusion criteria.

Subjects who were not county residents for ≥1 year, did not receive contrast for abdominal CT scan, CT image did not include the pancreas, and those without CT images available for review were excluded from the study. Subjects needed to be residents for ≥1 year to ensure they were true residents of the county and not temporary residents for treatment purposes.

eAppendix 2. Clinical and demographic data of the computed tomography cohort.

Clinical and demographic data entered in the study database included date of birth, sex, race, height, weight, history of acute pancreatitis, family history of PC, history of diabetes mellitus including date of the diagnosis, when available, smoking history, and presence of obstructive jaundice at the time of index imaging. Serum carbohydrate antigen (CA) 19-9, fasting blood glucose and HbA1c were also abstracted when available within 1 year of index CT imaging. The health record was manually reviewed for all study patients to identify future pancreatic resection and surgical pathology, development of PC, date of death and cause (related to pancreatic cancer, other, or unknown).

eAppendix 3. Categories of suspected/presumed intraductal papillary mucinous neoplasm based on a priori definitions.

These categories were defined as following: (1) *Suspected branch duct (BD) IPMN*: PCL >5 mm in diameter communicating with the main duct and/or cyst fluid analysis with carcinoembryonic antigen (CEA) >192 ng/mL (when measured) and/or multifocal cysts, at least one of which clearly communicated with the main pancreatic duct (MPD); (2) *Presumed BD-IPMN*: PCL without clear communication with MPD and/or CEA >192 ng/mL (when measured) and if clinically acted on, were treated as BD-IPMN; (3) *Suspected main duct IPMN*: segmental or diffuse dilation of MPD ≥5 mm without any obvious cause of pancreatic duct obstruction; (4) *Suspected mixed IPMN*: PCL meeting criteria for both main duct IPMN and BD-IPMN.

© 2023 de la Fuente J et al. *JAMA Network Open.*

eAppendix 4. Clinical and demographic data of the pancreatic cancer cohort.

Relevant demographic data included date of birth, sex, and race. Clinical stage of the PC at the time of diagnosis based on the exocrine pancreatic cancer tumor node metastasis staging American Joint Committee on Cancer/ Union for International Cancer Control 8th edition and relevant clinical and laboratory data at the time of diagnosis including: serum CA 19-9 level, smoking history, height, weight, and family history of PC. Treatment history and outcomes including date of last follow up, date of death, and cause of death (when available) were also recorded.

eAppendix 5. Pancreatic cancer imaging classification using a priori definitions to establish malignant transformation of intraductal papillary mucinous neoplasm.

(1) *Definitive IPMN-PC*: Nodules seen within pancreatic main duct AND/OR pancreatic mass arising within BD-IPMN; (2) *Probable IPMN-PC*: PCL involved/deformed by pancreatic mass AND/OR main duct dilation not explained by mass alone; (3) *Possible IPMN-PC*: PCL adjacent to pancreatic mass, but doubtful it originates from mass; (4) *Non-IPMN PC*: Pancreatic cystic lesion absent or present but remote from the pancreatic mass.

© 2023 de la Fuente J et al. *JAMA Network Open.*

eTable 1. Current Procedural Terminology (CPT) codes of abdominal computed tomography (CT) imaging used to develop the CT cohort.

CPT Code	Description
**74160**	Computed tomography, abdomen; with contrast material(s)
**74174**	Computed tomographic angiography, abdomen and pelvis, with contrast material(s), including noncontrast images, if performed, and image postprocessing
**74175**	Computed tomographic angiography, abdomen, with contrast material(s), including noncontrast images, if performed, and image postprocessing
**74177**	Computed tomography, abdomen and pelvis; with contrast material(s)

© 2023 de la Fuente J et al. *JAMA Network Open.*

eTable 2. Index codes used to collect the pancreatic cancer cohort.

Index code	Code Description
**ICD-10**	
C25.0	Malignant neoplasm of head of pancreas
C25.1	Malignant neoplasm of body of pancreas
C25.2	Malignant neoplasm of tail of pancreas
C25.3	Malignant neoplasm of pancreatic duct
C25.4	Malignant neoplasm of endocrine pancreas
C25.7	Malignant neoplasm of other parts of pancreas
C25.8	Malignant neoplasm of overlapping sites of pancreas
C25.9	Malignant neoplasm of pancreas, unspecified
Z85.07	Personal history of malignant neoplasm of pancreas
D01.7	Carcinoma in situ of other specified digestive organs
**ICD-9**	
157	MALIGNANT NEOPLASM OF PANCREAS
157.0	MALIGNANT NEOPLASM OF HEAD OF PANCREAS
157.1	MALIGNANT NEOPLASM OF BODY OF PANCREAS
157.2	MALIGNANT NEOPLASM OF TAIL OF PANCREAS
157.3	MALIGNANT NEOPLASM OF PANCREATIC DUCT
157.4	MALIGNANT NEOPLASM OF ISLETS OF LANGERHANS
157.8	MALIGNANT NEOPLASM OF OTHER SPECIFIED SITES OF PANCREAS
157.9	MALIGNANT NEOPLASM OF PANCREAS, PART UNSPECIFIED
V10.09	PERSONAL HISTORY OF MALIGNANT NEOPLASM OF OTHER SITES IN GASTROINTESTINAL TRACT (includes pancreas)
230.9	CARCINOMA IN SITU OF OTHER AND UNSPECIFIED DIGESTIVE ORGANS
**HICDA Code**	
01570110	NEOPLASM, MALIGNANT, PANCREAS, HEAD, PRIMARY
01578110	NEOPLASM, MALIGNANT, PANCREAS, NEC, PRIMARY
01578210	NEOPLASM, MALIGNANT, PANCREAS, BODY, PRIMARY
01578310	NEOPLASM, MALIGNANT, PANCREAS, NECK, PRIMARY
01578410	NEOPLASM, MALIGNANT, PANCREAS, TAIL, PRIMARY
01579110	NEOPLASM, MALIGNANT, PANCREAS, NOS, PRIMARY
01579111	TUMOR, ISLET CELL, PANCREAS, MALIGNANT
01579112	GASTRINOMA, PANCREAS, MALIGNANT
01579113	GLUCAGONOMA, MALIGNANT (PANCREAS)
01579114	CARCINOMA, GLUCAGON PRODUCING, CODE ALSO NEOPLASM MALIGNANT BY SITE (PANCREAS #)
01579115	SOMATOSTATINOMA
01579115	SOMATOSTATINOMA
01579116	INSULINOMA, MALIGNANT (PANCREAS)
34087110	HISTORY OF, MALIGNANCY, PANCREAS
**Berkson Code**	

X35832	CYST, MALIGNANT, PANCREAS
X35832	CARCINOMA, E.A.A. PANCREAS
X35832	CARCINOMA, E.A.A. INSULAR TISSUE
X35841	CARCINOMA, E.A.A. AMPULLA OF VATER
X35841	CARCINOMA, E.A.A. PAPILLA OF VATER
X35841	CARCINOMA, E.A.A. AMPULLA
X28922	SARCOMA, E.A.A. PANCREAS
X26822	TUMOR, ISLET-CELL
X26822	TUMOR, E.A.A. PANCREAS
X26822	INSULOMA

ICD: International classification of diseases; HICDA: Hospital international classification of diseases adapted.

eTable 3. Demographics and clinical characteristics of the computed tomography cohort

	IPMN (N=231)	No IPMN (N=1883)	p value
**Age at image**			**< 0.001**
Median (Q1, Q3)	77.8 (66.1, 84.7)	66.7 (57.4, 76.7)	
Mean (SD)	75.4 (12.1)	67.7 (11.8)	
**Sex**			**0.002**
Male	84 (36.4%)	890 (47.3%)	
Female	147 (63.6%)	993 (52.7%)	
**Race**			0.831
Asian	4 (1.7%)	51 (2.7%)	
Black	4 (1.7%)	38 (2.0%)	
White	215 (93.1%)	1728 (91.8%)	
Other/NA	8 (3.5%)	66 (3.5%)	
**Body Mass Index, kg/m^2 †**			**0.002**
Median (Q1, Q3)	26.0 (23.1, 29.3)	27.7 (24.2, 32.1)	
Mean (SD)	26.9 (5.6)	29.3 (11.4)	
**Smoking status***			0.128
Current	26 (11.4%)	260 (13.9%)	
Former	61 (26.6%)	550 (29.3%)	
Never	142 (62.0%)	1067 (56.8%)	
**Acute pancreatitis history**			0.738
Yes	10 (4.3%)	73 (3.9%)	
No	221 (95.7%)	1810 (96.1%)	
**Family history of pancreatic cancer**			0.404
Yes	8 (3.5%)	88 (4.7%)	
No	223 (96.5%)	1795 (95.3%)	
**Diabetes mellitus[#]**			0.143
No	177 (77.3%)	1550 (82.4%)	
LSDM	44 (19.2%)	257 (13.7%)	
NOD	7 (3.1%)	60 (3.2%)	
Unclassified DM	1 (0.4%)	15 (0.8%)	

IPMN: Intraductal papillary mucinous neoplasm; LSDM: Longstanding diabetes mellitus over 3 years since diagnosis; NOD: New onset diabetes, within 3 years of diabetes diagnosis

† Body mass index data was missing in 9 in the IPMN group and 97 in the No IPMN group

* Smoking Status was missing in 2 in the IPMN group and 6 in the No IPMN group

© 2023 de la Fuente J et al. *JAMA Network Open.*

Diabetes mellitus was missing in 2 in the IPMN group and 1 in the No IPMN group

© 2023 de la Fuente J et al. *JAMA Network Open.*

eTable 4. Demographics and clinical characteristics of the pancreatic cancer cohort

	IPMN-PC (N=31)	Non-IPMN-PC (N=284)	p value
**Age at diagnosis**			**< 0.016**
Median (Q1, Q3)	76.4 (70.9, 81.3)	72.3 (62.4, 80.1)	
Mean (SD)	76.9 (9.2)	71.3 (12.5)	
**Sex**			0.440
Male	14 (45.2%)	149 (52.5%)	
Female	17 (54.8%)	135 (47.5%)	
**Race**			0.562
Asian	0 (0.0%)	6 (2.1%)	
Black	0 (0.0%)	4 (1.4%)	
White	29 (93.5%)	264 (93.0%)	
Other	0 (0.0%)	3 (1.1%)	
Unknown	2 (6.5%)	7 (2.5%)	
**Body Mass Index, kg/m^2**			0.245
Median (Q1, Q3)	23.7 (21.7, 28.0)	25.9 (22.4, 29.2)	
Mean (SD)	25.2 (4.4)	26.4 (5.9)	
**Smoking status***			0.315
Current	1 (3.3%)	36 (12.7%)	
Former	15 (50.0%)	125 (44.2%)	
Never	14 (46.7%)	122 (43.1%)	
**Family history of pancreatic cancer[#]**			0.550
Yes	1 (3.3%)	17 (6.0%)	
No	29 (96.7%)	266 (94.0%)	
**CA 19-9**			0.697
Median (Q1, Q3)	110.0 (17.0, 2758.5)	387.5 (53.0, 2524.5)	
Mean (SD)	6677.4 (12941.3)	21461.1 (181301.5)	
**Stage at diagnosis**			**0.047**
I-III	20 (64.5%)	130 (45.8%)	
IV	11 (35.5%)	154 (54.2%)	
**Surgical resection**			**0.003**
Yes	14 (45.2%)	60 (21.1%)	
No	17 (54.8%)	224 (78.9%)	

© 2023 de la Fuente J et al. *JAMA Network Open.*

IPMN-PC: Malignant transformation of intraductal papillary mucinous neoplasm to pancreatic cancer;
CA 19-9: Carbohydrate antigen

* Smoking status was missing in 1 in the IPMN-PC group and 1 in the non IPMN-PC group

Family history was missing in 1 in the IPMN-PC group and 1 in the non IPMN-PC group

© 2023 de la Fuente J et al. *JAMA Network Open.*
