# Supplementary material for: Long-Term Outcomes and Risk of Pancreatic Cancer in Intraductal Papillary Mucinous Neoplasms
Source: JAMA Netw Open. 2023 Oct 17;6(10):e2337799. doi: 10.1001/jamanetworkopen.2023.37799 (PMC10582793; doi:10.1001/jamanetworkopen.2023.37799)
Supplement: Supplement 2. — Data Sharing Statement [file jamanetwopen-e2337799-s002.pdf]

Data Sharing Statement

de la Fuente. Long-Term Outcomes and Risk of Pancreatic Cancer With Intraductal Papillary Mucinous Neoplasms. *JAMA Netw Open*. Published online October 13, 2023. doi:10.1001/jamanetworkopen.2023.37799

Data

Data available: Availability of data and study material from a secure Mayo Clinic server is subject to approval from the authors and the Mayo Clinic Internal Review Board. Analytic methods are included in the main manuscript.
